# Weakly Supervised Occupancy Prediction Using Training Data Collected via Interactive Learning

**DOI:** 10.3390/s22093186

**Published:** 2022-04-21

**Authors:** Omar Bouhamed, Manar Amayri, Nizar Bouguila

**Affiliations:** 1 Concordia Institute for Information Systems Engineering (CIISE), Concordia University, Montreal, QC H3G1T7, Canada; omar.bouhamed@mail.concordia.ca; 2 G-SCOP Lab, Grenoble Institute of Technology, 38031 Grenoble, France; manar.amayri@grenoble-inp.fr

**Keywords:** deep learning, interactive learning, machine learning, occupancy prediction, time series

## Abstract

Accurate and timely occupancy prediction has the potential to improve the efficiency of energy management systems in smart buildings. Occupancy prediction heavily depends on historical occupancy-related data collected from various sensor sources. Unfortunately, a major problem in that context is the difficulty to collect training data. This situation inspired us to rethink the occupancy prediction problem, proposing the use of an original principled approach based on occupancy estimation via interactive learning to collect the needed training data. Following that, the collected data, along with various features, were fed into several algorithms to predict future occupancy. This paper mainly proposes a weakly supervised occupancy prediction framework based on office sensor readings and occupancy estimations derived from an interactive learning approach. Two studies are the main emphasis of this paper. The first is the prediction of three occupancy states, referred to as discrete states: absence, presence of one occupant, and presence of more than one occupant. The purpose of the second study is to anticipate the future number of occupants, i.e., continuous states. Extensive simulations were run to demonstrate the merits of the proposed prediction framework’s performance and to validate the interactive learning-based approach’s ability to contribute to the achievement of effective occupancy prediction. The results reveal that LightGBM, a machine learning model, is a better fit for short-term predictions than known recursive neural networks when dealing with a limited dataset. For a 24 h window forecast, LightGBM improved accuracy from 38% to 50%, which is an excellent result for non-aggregated data (single office).

## 1. Introduction

According to recent statistics [1], buildings account for 40% of the total energy usage in the world. Occupants’ behavior significantly influences that energy usage. In order to reduce energy consumption, using artificial intelligence and smart equipment, it is possible to control HVAC and lighting devices remotely [2,3,4,5]. However, on a daily basis, it is required to tune the HVAC system manually. Recent research works have shown that it is possible to save HVAC energy, by automating its control, in a given building via occupancy detection (i.e., detecting the presence or the absence of occupants inside the building and even inferring their number at a given point of time or within an interval) and prediction [6,7,8]. When provided accurate occupancy models, demand-driven control can utilize such information to coordinate real-time HVAC usage, reducing energy use and maintaining indoor thermal comfort in buildings. Compared to occupancy detection and estimation, occupancy prediction, which refers to predicting the number of occupants at a given time in the future or creating occupancy profiles given previous measurements, has received less attention in the literature. In this paper, we focus mainly on occupancy prediction using purely data-driven models which can learn from historical data and predict the occupancy based on previous related patterns.

Occupancy prediction is a challenging task due to the stochastic nature (i.e., mainly Markovian) of occupants’ behavior [9,10,11] and the difficulty to obtain training data. In recent years, an increasing number of researchers have used ML methods to create occupancy models. In [12], the behavior of occupants in a housing complex adjusting thermostat settings and heating system operations was investigated. Then, using machine learning methods such as clustering and decision trees, the occupant behavior pattern was determined. In [13], the authors developed a statistical machine learning framework, based on random forests, to predict heating load and cooling load of residential buildings with the objective of learning the occupants’ behavior patterns. In [14], using a complex sensor network, the authors investigated the relationship between ambient conditions and the number of occupants. CO${}_{2}$ and acoustic metrics have the strongest link with the number of occupants due to the huge number of open offices. Three machine learning algorithms (hidden Markov models, ANN, and SVM) were used to predict the occupancy schedule in a typical day during the test. Some other approaches have been based on the use of sensory data, use of electrical appliances, and water usage, and have deployed mainly machine learning (ML) techniques [15,16,17,18,19,20].

The recent prediction studies have shown that the performance of data-driven models depends mainly on three factors [21]: (1) quality of the training data used to learn the models, (2) selection of the input features, and (3) prediction algorithms used for model development. The goal of this paper is to develop a non-intrusive, privacy-preserving, and low-cost occupancy prediction unified framework, that simultaneously takes these three factors into account, via some features and information extracted from sensors such as temperature, power consumption, motion detection, humidity, and CO${}_{2}$ data. Different machine/deep learning techniques were considered for prediction. Machine/deep learning has attracted substantial attention from the research community and demonstrated great potential in many energy and building applications. Yet, its use to make accurate occupancy predictions requires large amounts of labeled data. However, labeling occupancy data is cumbersome since it generally needs the involvement of the occupants. In this work, this problem was tackled by proposing a labeling approach based on an interactive occupancy estimation technique that was previously proposed in [22]. Thus, the prediction framework will be mainly based on a mix of manually and probabilistically labeled training sets which can be viewed as a weakly supervised [23] prediction approach. To the best of the authors’ knowledge, it is the first time that a weakly supervised framework is proposed for occupancy prediction, i.e., the use of few but high-quality labeled data collected via the interactive learning approach to provide reliable future occupancy predictions.

The rest of this paper is structured as follows. Section 2 presents the developed occupancy prediction framework. Section 3 describes and discusses the extensive simulations and experimental results. Conclusions and potential future works are drawn in Section 4.

## 2. Occupancy Prediction Framework

The purpose of this section is to go over the occupancy prediction framework in depth. The section begins by presenting the overall prediction methodology, followed by a description of the case study under consideration. Finally, the used occupancy estimation approach to generate training data, which was previously proposed and successfully tested in [22], is briefly introduced.

### 2.1. Methodology

**Figure 1:**
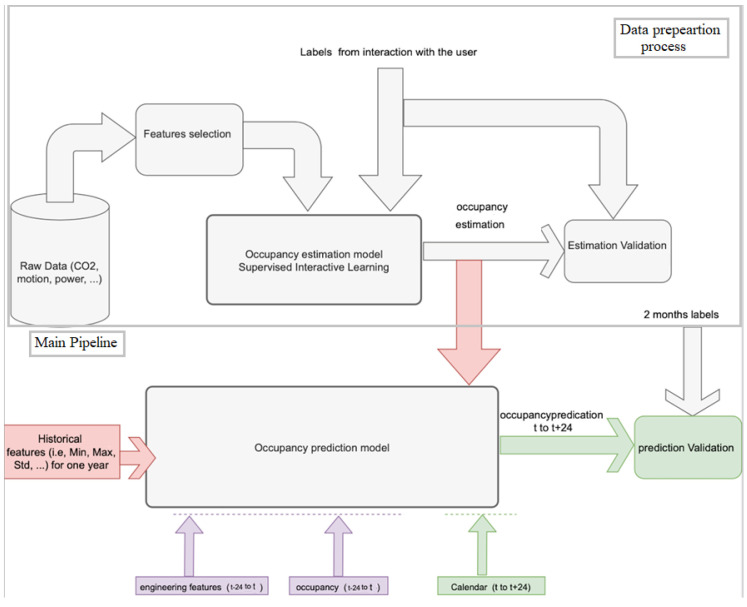
General occupancy prediction methodology. The following are the two primary processes: (1) the data preparation process through the generation of specific occupancy labels for existing unlabeled data, and (2) the main pipeline, which leverages the data created by the first step, as well as other extracted features, to estimate occupancy for various window sizes.

Due to occupancy’s stochastic nature, its short-term prediction for individual rooms remains a challenging task. This study aims to develop a concrete occupancy prediction model by applying machine/deep learning techniques. However, in order to reach the occupancy prediction part, a crucial process is required: the data preparation. Therefore, this study is mainly divided into two steps (as shown in Figure 1): (1) data preparation process and (2) occupancy prediction, the main pipeline.

The first step of occupancy label preparation is necessary due to the difficulty in collecting training data in the case of occupancy prediction problems which makes it challenging compared with load, weather, and solar energy forecasting [24], for instance. To address this issue, first, a feature selection process was carried out on the various raw data acquired by sensors to select the most relevant information. Thereafter, relying on the selected raw data, occupancy data were generated by applying a supervised learning model with an interactive learning approach that depends on a spread rate concept, to validate the quality of generated data, during one year in the office case study [22] (this part is illustrated by the upper part of Figure 1).

Thereafter, once the occupancy estimations are available (provided from the first step), we proceed with the next step: the establishment of the occupancy prediction model (the main pipeline). However, most of the time, occupancy measurements are insufficient to build an accurate and efficient prediction model for at least 24 h ahead of time; thus, to improve the prediction process, various prior knowledge and engineering features (e.g., the calendar, the office events such as meetings and presentations which most of the time show an increase in occupancy, the weather, and others such as the avg/minimum/maximum occupancy values for past days, etc.) were introduced.

### 2.2. Case Study

**Figure 2:**
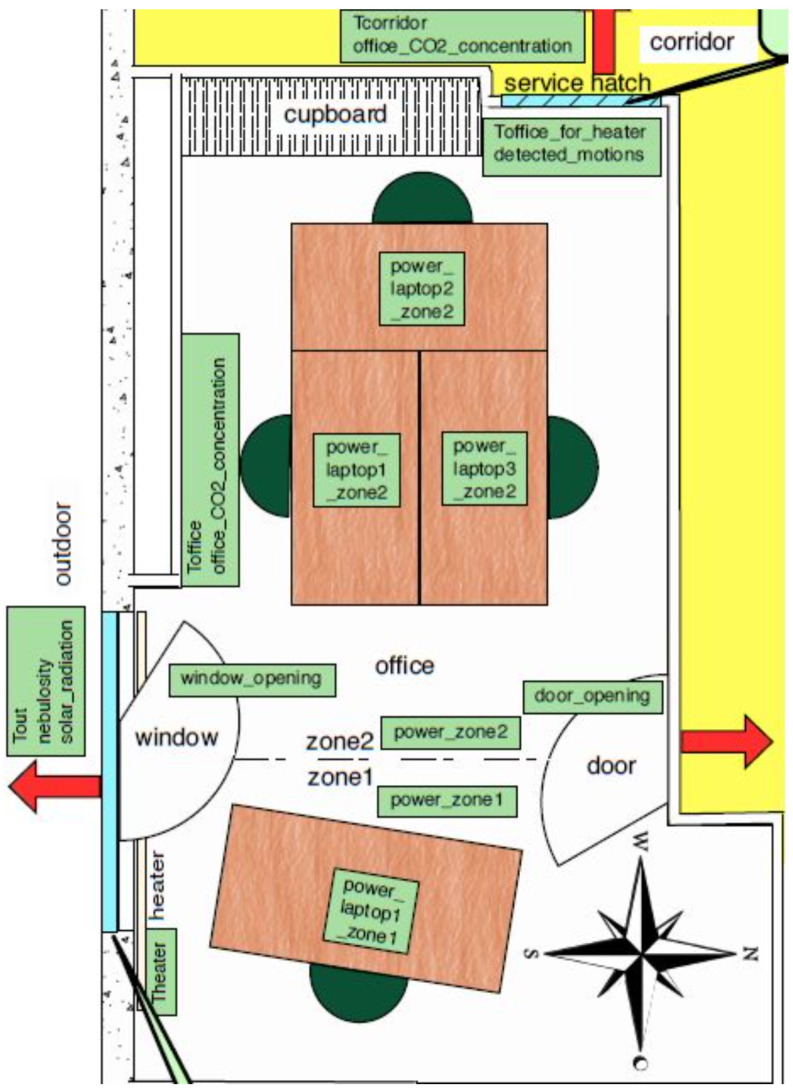
Office in Ense3, Grenoble Institute of Technology, building.

The testbed setup is at the Grenoble Institute of Technology as illustrated in Figure 2 and described in [25]. It is an office that accommodates a professor and three Ph.D. students. The office has regular visits during the week, with a lot of meetings and presentations. The setup includes different sensing networks that monitor indoor and outdoor temperature, humidity, motion detection, CO${}_{2}$ concentration, and acoustic pressure. It also contains a web application with a consolidated database for continual data retrieval from various sources. Statistical features were extracted from the raw sensory data. Then, the most relevant features were selected employing information gain [25]. The features *F* that will be used in this work are: motion counting, acoustic pressure (from microphone), power consumption, CO${}_{2}$ concentration, and door position.

### 2.3. Occupancy Estimation and Interactive Learning

For the case of supervised learning methods in general, and for the estimation of the number of occupants in particular, the problem of the acquisition of accurate target values arises, i.e., the labeling issue. Many of the known and efficient solutions in the literature are based on image processing, information extracted from camera feeds, which is still not acceptable in many locations when it comes to respecting occupants’ privacy. Only recently, a few works have proposed occupancy estimation approaches using non-intrusive sensors, with minimal impact on the persons involved. One of the most successful approaches, based on the concept of interactive learning, has been proposed in [22]. Interactive learning (IL) is a supervised learning methodology that involves exchanging information with the user to collect a training dataset related to a specific context [25]. IL, with a minimal number of interactions, i.e., asks, ensures the collection of good-quality data with accurate labels. This approach, proposed in [25], has been successfully applied to estimate the occupancy in office rooms, using different sensors and avoiding the use of cameras [22]. In addition, the concept of interactive learning allows us to evaluate and improve the quality of the database [26].

#### Interactive Learning Methodology

Interactive learning estimates the number of occupants by questioning occupants when relevant, limiting the number of interactions, and maximizing the information usefulness about the actual occupancy [22]. Occupancy estimation algorithms make use of information gathered from occupants as well as common sensors. The interactive learning approach is primarily dependent on interaction methodology to determine when it is necessary to ‘ask’ the occupants. The *ask* is a question displayed on a screen with its order, date, and time, i.e., Question 1, 05/09/2019 15:42:12 How many occupants in last 30 min? (0…7), while in a response area, there are different options, defined according to a minimum and a maximum possible number of occupants with a timeout of 3 h for each question. Four criteria have been taken into account to determine the interaction time. The first one is the density of the neighborhood, which is defined as the number of existing records (i.e., vectors of sensor features) in the neighborhood of a potential *ask* (i.e., interaction with the occupant). The second criterion is the classifier estimation error in the neighborhood of the potential *ask* which leads to the concept of neighborhood quality that was defined in [26] via a novel concept called spread rate. This methodology is based on the following. If the classifier estimation error is too high for a record, this record is removed from the neighborhood. However, an acceptable estimation error leads to updating the training set with the new record. The third criterion is based on the minimum class weight, which consists of the minimum acceptable number of records for each class. The fourth criterion of spread rate is a global measure of data quality (instead of counting the records, it checks how records are globally distributed). In the IL methodology, the number of occupants is then determined using supervised learning (ex., random forest, linear regression, etc.).

### 2.4. Prediction Models

The observations about the occupancy are collected at regular time intervals and are time series. The occupancy prediction problem can be stated as follows. Let $X^{t}$ denote the occupancy during the *t*th time interval. Given a sequence $\left\{X^{t}\right\}$ of observed occupancy data, t=1,2,…,T, that include the number of occupants $n^{t}$ and other information (e.g., calendar, sensor features, etc.), the problem is to predict the number of occupants $n^{t+\Delta }$ at time interval (t+Δ) for some prediction horizon Δ. In this paper, the following horizons in terms of hours were considered: Δ∈{1,12,24}. Predicting the future occupancy is based on previously collected time series using several well-known approaches from the literature, namely support vector regression (SVR), multi-layer perceptrons (MLPs) [27], and recurrent neural networks (RNNs) [28]. In particular, different RNN variants, namely long short-term memory (LSTM), CNN-LSTM, and gated recurrent unit network (GRU), were considered [28]. In addition, an investigation was carried out to explore light gradient boosting machine (LightGBM) which is a gradient boosting open-source framework for gradient boosted machines [29].

#### 2.4.1. Multi-Layer Perceptron (MLP)

The field of artificial neural networks (ANNs) is often referred to as neural networks or multi-layer perceptrons, the “vanilla” neural networks. The power of neural networks comes from their ability to deduct the relation between the inputs and outputs, in other words, comprehend the representation in the training data and how to best relate it to the output variable to predict, which is in this case the occupancy.

While a single layer perceptron can only learn linear functions, an MLP is able to learn both linear and non-linear functions. At each layer, the neurons transform their inputs $x_{i}$ by calculating a weighted sum *z* over them ($w_{i}$: weights, b: bias), and then this transformation is subjected to a non-linear function ϕ, known as an activation function, in order to obtain an intermediate state *a*. Simply put, an MLP can be defined by the weights between its layers of neurons, the output of which is mostly computed using a non-linear function. MLPs are popularly known as universal function approximators. They are capable of learning weights that map the input to an output.

(1) $$\begin{array}{c} z=\sum_{i}w_{i}\cdot x_{i}+b. \end{array}$$

(2) $$\begin{array}{c} a=\phi (z). \end{array}$$

However, this simple architecture faces a lot of challenges, such as the vanishing and exploding gradient. An issue is related to the back-propagation algorithm, which is a technique used to update the weights of a neural network by calculating the gradients. In the case of an extremely deep neural network (a large number of hidden layers), the gradient vanishes or bursts as it propagates backward, resulting in disappearing and exploding gradients. Another major defect for this type of architecture is that the latter cannot capture sequential information in the input data which is required for dealing with sequence data which makes it not a great fit for time series forecasting.

#### 2.4.2. RNN: LSTM and GRU and bi-LSTM

A looping constraint on the hidden layer of an ANN turns it into an RNN. That is to say that an RNN has an additional looping constraint on the hidden layer than the simple ANN. This addition gives the RNN a sense of time context which ensures that sequential information is captured in the input data, hence overcoming one of the ANN’s major issues. However, this was not sufficient to suppress the other problem, the vanishing gradients. Therefore, new models, such as long short-term memory (LSTM) and gated recurrent units (GRUs), were introduced. For instance, LSTM relies on a new state called cell state and has a constant error carousel (CEC) which allows the error to propagate back without vanishing. The cell state and its many gates are at the heart of LSTMs. The latter serves as a carrier for relative information to transfer it all the way down the sequence chain. It acts more like the network’s “memory”, carrying meaningful information throughout the sequence’s processing. As a result, knowledge from earlier time steps can be used in later time steps, lessening the impact of short-term memory. As the cell state travels, information is added or deleted from the cell state via gates. The gates are neural networks that determine which information remains in the cell state. During training, the gates have the capability to learn which information is relevant to keep and which to discard.

As previously stated, the LSTM contains complex dynamics that allow it to easily “memorize” information over a long period of time. The “long-term” memory is kept in a vector of memory cells $c_{t}\in R^{n}$. The LSTM architecture used in our experiments is given by the following equations [30]:

$$\begin{array}{cc} \; & i_{t}=\sigma \left(W_{i}\left[h_{t-1},x_{t}\right]+b_{i}\right),\\ \; & f_{t}=\sigma \left(W_{f}\left[h_{t-1},x_{t}\right]+b_{f}\right),\\ \; & o_{t}=\sigma \left(W_{o}\left[h_{t-1},x_{t}\right]+b_{o}\right),\\ \; & g=\tanh(w_{c}\left[h_{t-1},x_{t}\right]+b_{c}),\\ \; & c_{t}=f⊙c_{t-1}^{l}+i⊙g,\\ \; & h_{t}=o⊙\tanh\left(c_{t}\right), \end{array}$$

where $i_{t}$ represents the input gate, $f_{t}$ the forget gate, $o_{t}$ the output gate, σ the sigmoid function, $w_{x}$ the weight for the respective gate (*x*) neurons, $h_{t-1}$ the output of the previous LSTM block (at timestamp t−1), $x_{t}$ the input of current timestamp, $b_{x}$ the bias for the respective gates (*x*), *g* the candidate for cell state at timestamp (*t*), and $c_{t}$ the cell state (memory) at timestamp (*t*).

GRU shares similar characteristics with LSTM. To manage the memorizing process, both algorithms include a gating mechanism. GRU, on the other hand, is less complicated than LSTM and substantially faster to calculate. Later on, other architectures were introduced, for example, bidirectional-LSTM, which is unlike LSTM that only preserves information from previously processed inputs, and Bi-LSTM runs the inputs in both ways (from past to future and from future to past), which allows it to preserve information from the past and future.

#### 2.4.3. LightGBM

According to [29], LightGBM is a revolutionary gradient boosted decision tree (GBDT) method that arose as a result of the previous implementation of GBDT’s lack of efficiency and scalability while dealing with high-dimensional features and large data size.

LighGBM is a high-performance gradient boosting framework that utilizes gradient-based one-side sampling (GOSS) and exclusive feature bundling (EFB) to enhance computational efficiency without sacrificing accuracy. GOSS is used to split the optimal node by calculating variance gain, whereas EFB can speed up the GBDT training process by grouping numerous exclusive features into fewer dense features [31].

**Figure 3:**
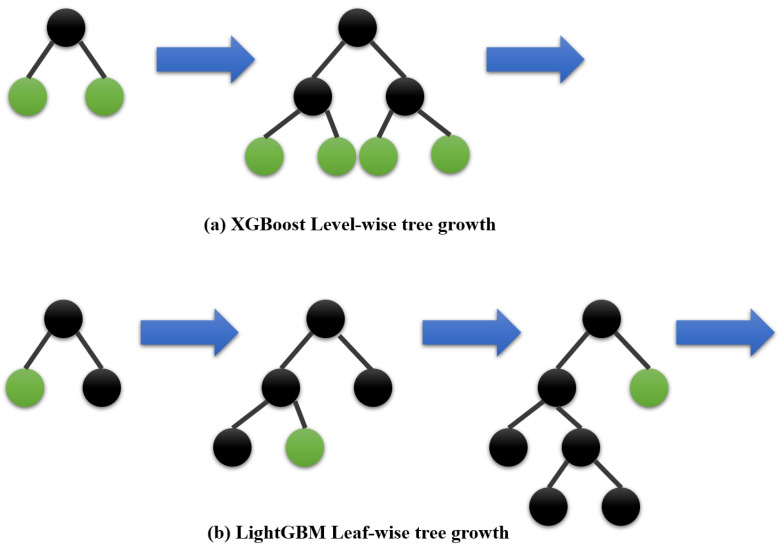
XGBoost vs. LightGBM tree growth.

As illustrated in Figure 3, LightGBM develops trees vertically, whilst other algorithms do that horizontally, indicating that LightGBM grows trees leaf-by-leaf, whereas other algorithms grow trees level-by-level, for instance, XGBoost. LightGBM grows the leaf with the greatest delta loss. The leaf-wise technique minimizes loss more than the level-wise procedure while extending the same leaf.

## 3. Experimental Results

This section is divided into three sections, each of which is strongly influenced by the nature of the estimated data. A discrete occupancy prediction study is presented in the first section, where the problem is initially treated with only two classes (0: absence of occupant and 1: the presence of occupant(s)), leading to the second section with three classes (0: absence of occupant, 1: the presence of one occupant, and 2: the presence of more than one occupant). In the second part, the problem’s complexity is increased by transforming it into a regression problem rather than a classification problem (continuous data, i.e., the average number of occupants per hour in the office, instead of discrete). The data were originally collected every 5 min, thus we have 5 min sampled data. Judging that working with hourly sampled data is more efficient (reduced input size), the necessary changes were performed (resampling the data at a 1 h rate by averaging the sensors reads per hour).

### 3.1. Additional Feature Extraction

Before diving into the prediction problem, as mentioned before, relying only on occupancy measurements is not enough to feed the prediction model with the necessary information to produce accurate outputs, hence, an investigation of the usage of different features was made to improve the process.

#### 3.1.1. Time Features

**Figure 4:**
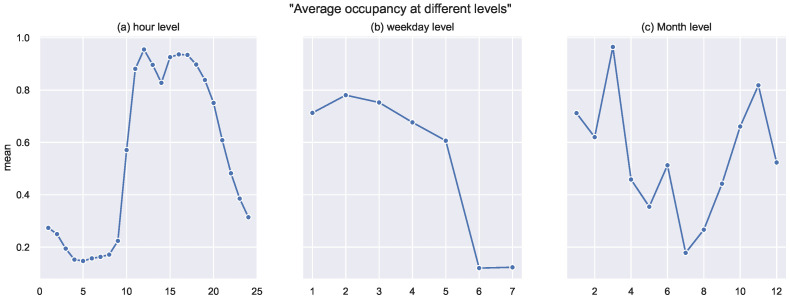
Average occupancy at different time levels: (**a**) hourly level, (**b**) daily level, and (**c**) monthly level.

Date and time components (month, day of week, and hour) were extracted from the available date–time variable. Such features might seem simple, but in reality, they provide the model with significant information. For instance, as depicted in Figure 4a, it is clear that the highest occupancy levels are spotted at the hour interval between [10 a.m., 9 p.m.]. In Figure 4b, the highest occupancy levels were scored on the weekdays as, for the weekend, hardly any occupancy was detected. Such features help the prediction model to be more aware of the temporal circumstances.

#### 3.1.2. Calendar Features

The office calendar is directly connected with the presence of occupants and their activities inside the office which makes calendar features an interesting addition to improve the performance of the prediction model. Luckily, such information could be extracted from the Gmail calendar of the main office, which contains all office events (meetings, presentations, etc.) and their starting and ending times. Requests were written in a Python function to decode the calendar information to be introduced in a data frame that has the same structure as the occupancy data frame to be able to link.

**Figure 5:**
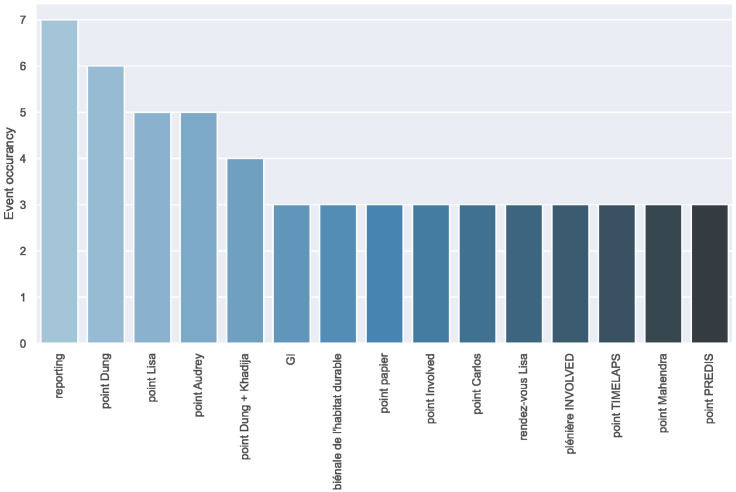
The most frequent office events and their counts.

Figure 5 shows a clear look at the event repetition, where the maximum occurrence is seven times (occurrence) for the event called “reporting” and then most recurrent events are the ones with “point” in their names; these events were coinciding with strong occupancy in the building. After further analysis, it seemed that the calendar events could be divided into two categories based on the occupancy levels of the events. Accordingly, in case of the occurrence of an event, the events with “point” or “reporting” were grouped as a category referred to as “g2” and all the remaining events were embedded as category “g1”, otherwise, in case there was no event, the sample was reported as category “g0”.

**Figure 6:**
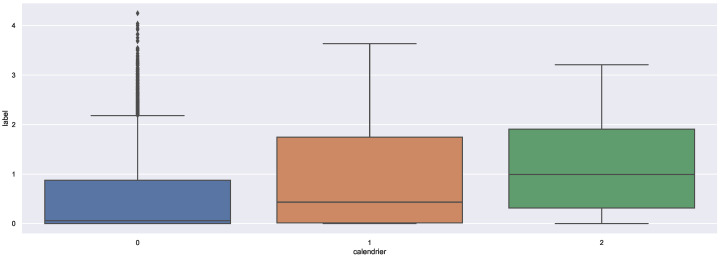
Box-plots of the occupancy as a function of calendar categories.

As shown in Figure 6, compared to the events in “g2” which match strongly with the occupancy, the “g1” events have an inferior impact on the occupancy in the office. Still, the presence of an event, whether from “g1” or “g2”, has a great influence on the occupancy level in the office.

The time and calendar features were both used as lag features (about the historical data) and for the lead features (for the time horizon to be predicted).

### 3.2. Discrete Prediction

#### 3.2.1. Binary Occupancy Estimation

The first step in validating the prediction framework is to use an interactive learning approach. Four criteria are used to interact with the user, as detailed in [22]: (1) data quality (spread rate), (2) density of the neighborhood, (3) minimum class weight, and (4) classifier estimation error.

Six relevant features are used in this experiment as input to the estimation model ((1) motion counting, (2) acoustic pressure, (3) power consumption, (4) CO${}_{2}$ concentration, (5) door position, (6) calendar). Considering the case study presented in Section 2.2, the real-time system was launched for one year. As mentioned previously, for the case of binary prediction, the output of the estimated occupancy data should follow the prediction objective. In other words, the data should consist of just two classes. Different experiments have been done and three methods have been considered and compared for occupancy estimation with interactive learning, namely logistic regression, support vector machine (SVM), and random forest, using just two occupancy levels. According to the results in Table 1, which shows the quantitative measures for the three models, it is clear that all three models provided great results, but random forest performs the best.

#### 3.2.2. Binary Occupancy Prediction

**Figure 7:**
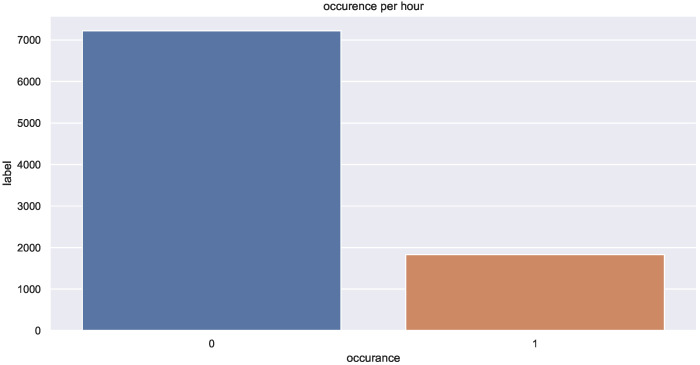
Binary data distribution.

The LightGBM classifier was chosen as a baseline model for this section. The advantage of LightGBM is that even with quick training, it provides accurate results. Hyperparameter tuning was also employed with a 6-fold cross-validation that enabled us to find the best LightGBM model. Coupled with the additional features, the outputs of the estimation model were fed to the prediction model. The preliminary results were not satisfying due to the fact that the data are unbalanced (class 0 covers more than 80% of the total dataset samples) as shown in Figure 7.

Therefore, more advanced configurations were explored to overcome such challenges. One of the solutions that considerably improved the preliminary results was the use of the classes’ weights. When computing the loss function, classes’ weights are employed to avoid the model from favoring the major class. If one class dominates the dataset, the model will be biased to learn that class better, because the loss is primarily determined by the model’s performance on that dominant class. Weighting involves raising a class contribution to the loss function. As a result, the contribution of the gradient for that specific class will be greater as well. So, instead of using the target values, the gradient and Hessian were multiplied by the weights, which are calculated based on the distribution of the classes.

The final results for 1/12/24 h ahead in time are summarized in Table 2, where a comparison is carried out between the actual occupancy values with the ones provided by the LightGBM model. It is shown that the prediction model learned how to efficiently predict class 0, the dominant class, and also has decent results for the minor class 1 predictions for the different window sizes, especially for the 1 h prediction, where the overall precision reached 94%.

**Figure 8:**
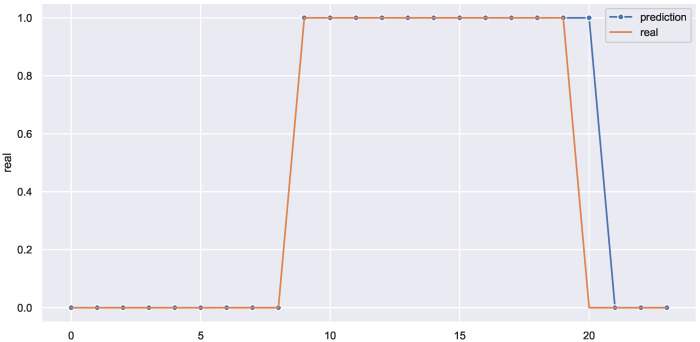
The framework prediction versus reality (24 h ahead) for the case of binary data (0 or 1).

The framework performance could be validated by the use case displayed in Figure 8 which shows a 24 h ahead prediction of one of the weekdays. The model successfully predicted the absence and presence of occupants in the office for almost all 24 h, except for hour 20, when it incorrectly predicted the presence of the occupant. Following further investigation, it appears that the model is lacking when it comes to accurately identifying the time of departure of occupants after lengthy periods of presence which affected the metrics shown previously in Table 2, for the 24 h ahead predictions.

#### 3.2.3. Multi-Level Occupancy Estimation

For the case of multi-level occupancy prediction, the complexity of the problem was increased by adding another level of occupancy. As a result, we have a total of three classes (0: absence of occupant, 1: the presence of one occupant, and 2: the presence of more than one occupant). This change made the estimation part a bit trickier, as evidenced by the results in Table 3.

**Figure 9:**
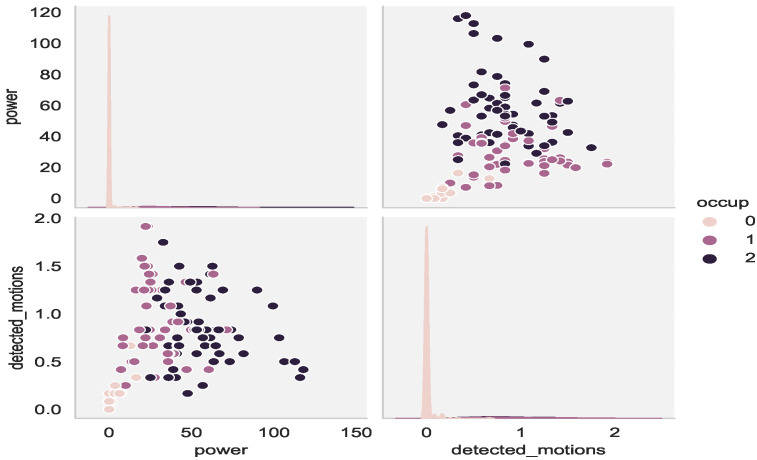
Pair-plot of power vs. detected_motions (avg per hour).

The performances of the three chosen models, in the case of multi-level occupancy, were inferior to those with binary data. As shown in Figure 9, finding the boundaries to accurately separate the two classes is a bit complicated, especially for classes 1 and 2.

#### 3.2.4. Multi-Level Occupancy Prediction

**Figure 10:**
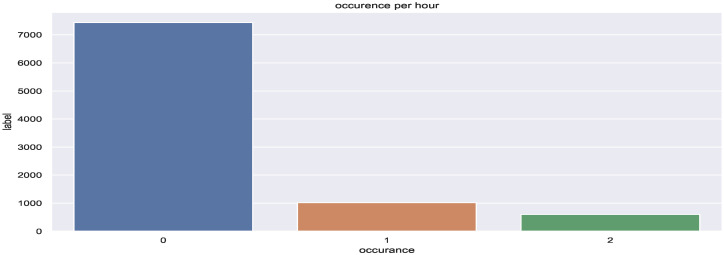
Multi-level data distribution.

As mentioned previously in the case of binary data, we faced a problem of unbalanced data where class 0 is dominant, and dividing the occupancy class into two levels, as shown in Figure 10, raised the difficulty bar for the prediction framework.

Table 4 summarizes the final results for the 1/12/24 h ahead in time predictions. It is demonstrated that the prediction model learned how to efficiently predict the dominant class, class 0, but the results for the occupancy levels were unsatisfactory. As shown in Figure 9 and Table 4, despite effectively learning how to distinguish absence from presence of occupants, the framework had difficulty specifying the level of occupants, i.e., the one or more occupants in the office.

**Figure 11:**
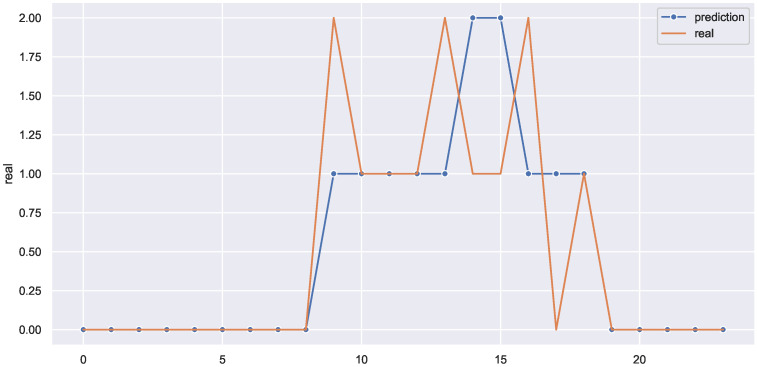
The framework predictions for multi-level occupancy versus reality (24 h ahead).

This could be validated by the use case displayed in Figure 11, which shows a 24 h prediction of one of the weekdays. The model accurately predicted the absence and presence of occupants in the office for almost the entire 24 h, but it failed to predict the correct number of occupants for a couple of hours. For example, instead of predicting two, the framework predicted one in hours 9, 13, and 16, and in hours 14 and 15, the framework output was two instead of one.

### 3.3. Continuous Occupancy

In this part, instead of the use of discrete data as in the previous part, continuous data were used. In other words, the problem was transformed from a classification one into a regression one.

#### 3.3.1. Continuous Occupancy Estimation

**Figure 12:**
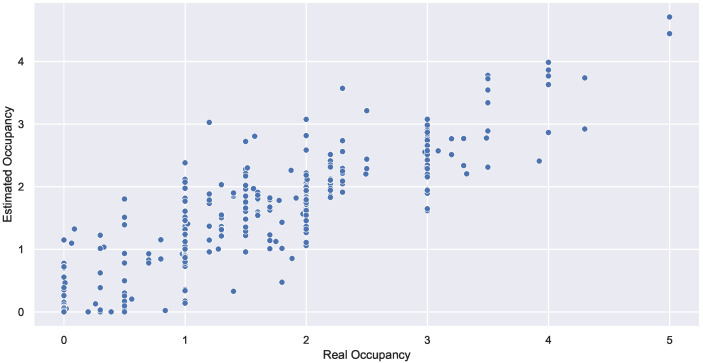
Estimated occupancy and real occupancy when considering random forest.

Several experiments were conducted, and three methods for occupancy estimation with interactive learning were considered and compared: linear regression, a gradient boosting regressor, and a random forest regressor with five occupancy levels. Furthermore, two evaluation measures were used: (1) mean squared error (MSE), a risk metric corresponding to the expected value of the squared (quadratic) error or loss, and (2) R2 score (R2S), a coefficient of determination (R2) between 0 and 1, with 1 being the best value. Table 5 displays the quantitative measures, while Figure 12 displays the graphical evaluations of the random forest regressor, which outperforms the gradient boosting regressor and linear regression.

**Figure 13:**
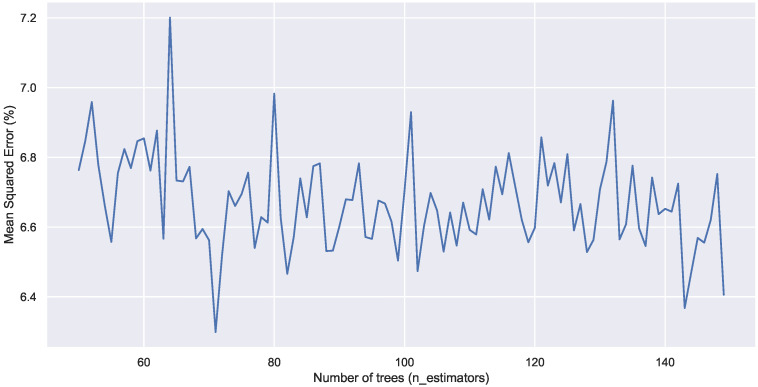
Mean squared error as a function of the number of trees.

An important parameter in the random forest regressor model is the number of trees in the forest. In the presented results, this parameter was set by default to 100. Figure 13 depicts the estimation error as a function of the number of trees and shows that changing the number of trees did not significantly reduce the error.

When compared to the other two methods, the random forest regressor produces the best results. It took 21 requests for training data to create an acceptable estimator. Table 6 shows how the 21 questions are distributed across the days using the random forest regressor.

#### 3.3.2. Continuous Occupancy Prediction

**Figure 14:**
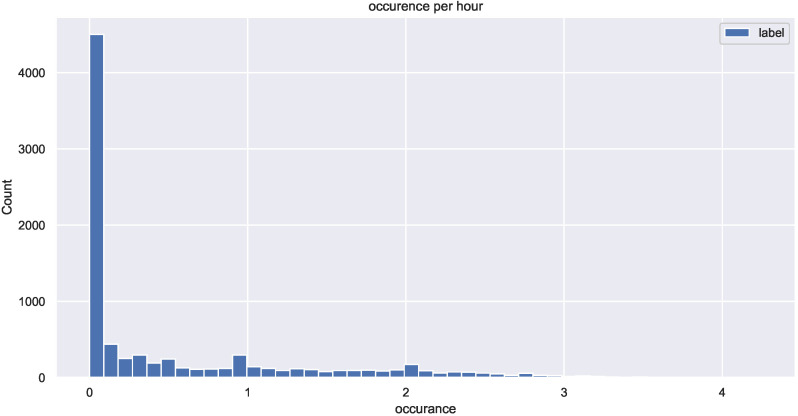
Continuous data distribution (avg number of occupants per hour).

Occupancy estimation using the random forest regressor and interactive learning provides us with accurately labeled occupancy data to use in the prediction models with an average error of 0.06. In this part of the study, different known algorithms were tested to visualize the impact of the additional features on the efficiency of the models. For this study, the occupancy data were used as they are, i.e., continuous data, as seen in Figure 14. The results for the prediction of the three-time windows (1 h/12 h/24 h ahead) are summarized in Table 7.

**Figure 15:**
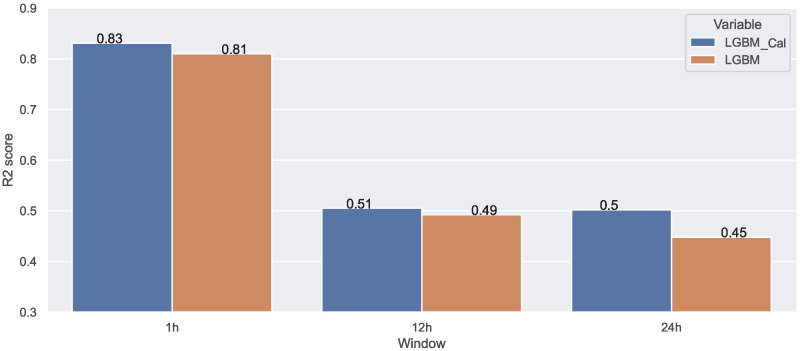
Additional features’ impact on LightGBM predictions.

As shown in Table 7, although the prediction results were close due to the lack in terms of data, LightGBM outperformed the other algorithms, especially for the 12 h and 24 h ahead predictions. As mentioned previously, LightGBM has the benefit of being able to achieve accuracy with less training time. Furthermore, we decided to investigate the impact of the additional features on the LightGBM performance. As shown in Figure 15, the added features improved the prediction, yet the results were not so satisfying for the 12 h/24 h prediction compared to the ones obtained while using discrete data, as expected. After all, the use of discrete data instead of continuous ones would decrease the complexity of the problem and consequently improve the final results which makes sense since there is less fluctuation in the data.

## 4. Conclusions

The building sector has become one of the largest energy consumers in the world. HVAC systems are responsible for most of the energy consumption. With the popularity of smart buildings, a huge amount of data could be collected and analyzed. These data can reflect and be used to infer very useful information and hidden knowledge that could be used for estimation (e.g., occupancy estimation) and prediction (e.g., occupancy prediction) tasks. The focus of this paper was building an occupancy prediction framework which holds great benefits for building control systems to provide comfort to occupants while saving energy. The proposed solution allows an efficient prediction of the number of occupants in an office relying on the data collected using the interactive approach as well as additional features extracted from the official calendar and event schedules. In the case of the use of binary data, it is shown that the proposed framework, based on LightGBM, can achieve satisfactory performance for different window sizes (1/12/24 h ahead), reaching a precision of 86% for 24 h ahead prediction. As for the case of continuous one, extensive simulations show that using non-aggregated data is difficult, particularly when the forecast window size is increased, and that this is attributable to the nature of data (not as smooth as the aggregated data). Recurrent neural network-based models such as LSTM, CNN LSTM, and GRU can produce good results for 1 h prediction, but their performance degrades as the window size is increased, with an average accuracy of 37% for 24 h prediction. LightGBM marginally suffers from the same dilemma, nevertheless, it achieves the best results among all the discussed models where the accuracy increases from 37% to 50%, which is a good result dealing with these kinds of data. Regardless, we believe that apart from the use of information extracted from the office calendar, it would be worthwhile to investigate the association between occupancy prediction and load forecasting. Future research could look into the possibility of using future load prediction as one of the additional features to improve the framework’s performance. Another intriguing concept to investigate is using an interactive approach in the occupancy prediction phase, i.e., involving the user-end in the prediction phase as well. To ensure reproducibility of the results by the research community and a potential future improvement of the framework by other researchers, the complete source code is provided in the following repository: https://github.com/OmarBouhamed/Occupancy_pred.

## Figures and Tables

**Table 1:** Estimation results.

ML-Algo	Precision (%)	Recall (%)	F1-Score (%)
**SVM**	95.17	92.99	94.03
**Logistic regression**	99.25	96.88	98.01
**Random forest**	100.0	100.0	100.0

**Table 2:** Binary prediction results.

	Precision (%)	Recall (%)	F1-Score (%)
**1 h window**			
**0: absence of occupants**	97.06	97.78	97.42
**1: presence of occupants**	95.71	94.37	95.04
**Overall (macro avg)**	90.06	90.42	90.24
**12 h window**			
**0: absence of occupants**	94.41	93.75	94.08
**1: presence of occupants**	85.71	87.10	86.40
**Overall (macro avg)**	90.06	90.42	90.24
**24 h window**			
**0: absence of occupants**	91.60	92.48	92.04
**1: presence of occupants**	81.12	79.19	80.14
**Overall (macro avg)**	86.36	85.84	86.09

**Table 3:** Estimation results.

ML-Algo	Precision (%)	Recall (%)	F1-Score (%)
**SVM**	79.0	76.21	75.16
**Logistic regression**	94.38	85.19	87.51
**Random forest**	95.12	93.4	92.0

**Table 4:** Multi-level prediction results.

	Precision (%)	Recall (%)	F1-Score (%)
**1 h window**			
**0: absence of occupants**	97.06	93.62	95.31
**1: presence of 1 occupant**	66.07	86.05	74.75
**2: presence of more than 1 occupant**	64.29	40.91	50.00
**Overall (macro avg)**	75.81	73.52	73.35
**12 h window**			
**0: absence of occupants**	93.60	91.99	92.79
**1: presence of 1 occupant**	55.09	68.56	61.09
**2: presence of more than 1 occupant**	57.61	39.85	47.11
**Overall (macro avg)**	68.76	66.80	67.00
**24 h window**			
**0: absence of occupants**	93.00	91.61	92.30
**1: presence of 1 occupant**	42.37	53,76	47.39
**2: presence of more than 1 occupant**	52.19	39.53	44.99
**Overall (macro avg)**	62.52	61.63	61.56

**Table 5:** Estimation results.

ML-Algo	MSE	R2S
**Linear regression**	0.136	0.82
**Gradient boosting**	0.084	0.89
**Random forest**	0.063	0.91

**Table 6:** Number of asks each day.

Days	1	2	3	4	5	6	7	8	9	10
**Number of asks**	10	4	0	2	2	0	2	1	0	0

**Table 7:** R2 score for different ML approaches.

ML-Regressor	1 h	12 h	24 h
**LightGBM**	0.831	0.505	0.502
**LSTM**	0.814	0.360	0.380
**CNN_LSTM**	0.813	0.367	0.371
**Bidirectional_LSTM**	0.817	0.363	0.378
**GRU**	0.805	0.370	0.318
**MLP**	0.785	0.330	0.370
**Regression**	0.800	0.400	0.162

## Data Availability

Not applicable.
